# Association of Soluble B7-H4 and Circulating Tumor Cells in Blood of Advanced Epithelial Ovarian Cancer Patients

**DOI:** 10.3389/fonc.2021.721067

**Published:** 2021-10-29

**Authors:** Pawel Mach, Rainer Kimmig, Sabine Kasimir-Bauer, Paul Buderath

**Affiliations:** Department of Gynecology and Obstetrics, University Hospital Essen, Essen, Germany

**Keywords:** epithelial ovarian cancer, sB7-H4, circulating tumor cells (CTC), tumor micro-environment, biomarker

## Abstract

**Introduction:**

Epithelial ovarian cancer (EOC) is the deadliest gynecologic malignancy worldwide. Reliable predictive biomarkers are urgently needed to estimate the risk of relapse and to improve treatment management. Soluble immune-checkpoints in EOC are promising molecules serving as prognostic biomarkers accessible *via* liquid biopsy. We thus, aimed at elucidating the role of sB7-H4 in EOC.

**Material and Methods:**

We analyzed soluble serum B7-H4 (sB7-H4) using ELISA and circulating tumor cells (CTCs) in blood applying the AdnaTest *OvarianCancer* in 85 patients suffering from advanced EOC. Findings were correlated with clinical parameters as well as survival data.

**Results:**

sB7-H4 was detectable in 14.1% patients, CTCs in 32.9% patients and simultaneous presence of CTCs and sB7-H4 was found in 7% patients, respectively. Although no association between sB7-H4 and CTC could be documented, each of them served as independent predictive factors for overall survival (OS).

**Conclusion:**

sB7-H4 and CTCs are independent prognostic biomarkers for impaired survival in EOC. As they are easily accessible *via* liquid biopsy, they may be of potential benefit for the prediction of therapy response and survival for EOC patients.

## Introduction

Epithelial ovarian cancer (EOC) is the deadliest gynecologic malignancy worldwide (1). In Germany, EOC is responsible for 3.2% of all malignant neoplasms and 5.3% of all cancer deaths (2). Despite a high response rate for initial therapy, most patients eventually relapse (3), leading to a relative 5-year survival of only 41 % in 2014 (2). Reliable predictive biomarkers are urgently needed to estimate the risk of relapse and improve treatment management. In this regard, it has already been demonstrated that the characterization of disseminated tumor cells (DTCs) in the bone marrow (BM) and circulating tumor cells (CTCs) in peripheral blood has identified stem cell like DTCs and CTCs, tumor cells in epithelial-mesenchymal transition (EMT) as well as resistant cells, all associated with a worse outcome and clinical platinum resistance (4–7). Interestingly, we were able to show that immunotherapy based on the intraperitoneal trifunctional bispecific antibody Catumaxomab was successful in the elimination of DTCs and CTCs in patients with advanced EOC (8). Thus, the tumor microenvironment might play a crucial role in tumor control and tumorigenesis of these patients.

In recent years, the role of the immune system in EOC has gathered increasing attention. Several mechanisms of immune-escape have been identified in malignant tumors, including the aberrant expression of immune costimulatory ligands, such as those of the B7 family, namely programmed cell-death protein 1 (PD-1) (also known as B7-H1) and its ligands, programmed cell-death ligand 1 and 2 (PD-L1 and PD-L2). In our recent study, we show that decreased serum levels of PD-L2 were associated with poor survival and platinum-resistance, as well as the presence of DNA excision repair protein ERCC+CTC, indicating the significance of the organism’s immune-response for the prevention of tumor-cell spread and the efficacy of platinum-based therapy (9).

B7-H4 is another member of the B7-family, which is highly expressed in various types of neoplasms including ovarian, endometrial, or breast cancer (BC). Its expression correlates with the progression of the disease and a poor prognosis in many cases (10, 11). Moreover, Liang et al. show that B7-H4 is more often expressed in high-grade serous EOC (12). Evaluating a serum-based, multi parametric biomarker panel in OC patients, Oikonomopoulou et al. found that high levels of B7-H4 were associated with worse overall survival (13). B7-H4 has a membrane-bound and soluble form (sB7-H4), which has also been detected in the serum of patients suffering from cancer with its expression closely related to progression and prognosis (14). However, the source and function of sB7-H4 are still unknown.

The hypothesis that immune cells in the bloodstream can expand the metastatic potential of CTCs has recently been confirmed in BC (15). Based on these findings, we hypothesize that simultaneous measurements of CTCs and sB7-H4 could help to better understand the complex interactions between the tumor microenvironment and the immune response in EOC. This study aimed to investigate the serum levels of sB7-H4 and CTCs in patients with advanced EOC and to analyze its relationship to prognosis and clinicopathological features.

## Material and Methods

### Cohort Characteristics

We analyzed data from 85 patients suffering from advanced EOC. All patients underwent primary debulking surgery including hysterectomy, bilateral salpingoovarectomy, omentectomy, peritonectomy and - in the case of macroscopic complete resection - systematic pelvic and paraaortic lymphadenectomy. Adjuvant treatment consisted of six cycles of chemotherapy with carboplatinum AUC5 and paclitaxel 175 mg/m² i.v. d1 q3w intravenously. Since 2012, patients presenting at FIGO stage IIIB or higher have been offered an additional adjuvant therapy with the antiangiogenetic antibody Bevacizumab 15 mg/kg i.v. d1 q3w for a total of 15 months. Patients were diagnosed between 2007 and 2016 at the university hospital in Essen, Germany. Clinical data were acquired from patient charts and the hospital’s clinical information system. The study was approved by the local ethics committee of the University of Duisburg-Essen (Essen 05-2870 and 17-7859).

Patients recurring within six months after the end of the adjuvant platinum therapy were considered platinum-resistant. Disease-Free Survival (DFS) was calculated from the date of initial diagnosis to relapse.

An overview of patients’ characteristics is given in **Table 1**.

**Table 1:** Clinical data of patients with advanced EOC at the time of primary diagnosis.

	Total n = 85 (%)	p-value
FIGO stage		
III	61 (71.8)	0.001
IV	24 (28.2)	
Grading		
1 and 2	24 (29.3)	0.0002
3	58 (70.7)	
Nodal status		
Node negative	24 (37.5)	0.04
Node positive	40 (62.5)	
Platinum sensitivity		
Sensitive	50 (84.7)	0.0001
Resistant	23 (27.1)	
Resection status		
Complete resection	39 (48.1)	0.73
Incomplete resection	42 (51.9)	
Recurrence		
No	29 (37.7)	0.03
Yes	48 (62.3)	

### Blood Serum Collection and Measurement of sB7-H4

Soluble (s) B7-H4 serum levels were analyzed preoperatively using the sandwich Enzyme-Linked Immunosorbent Assay Kit (Cusabio, Cologne, Germany) according to the manual instructions. For ELISA measurement, 100 µl undiluted serum samples and control samples were dispensed into wells coated with an antibody specific for B7-H4 and incubated for two hours at 37°C. Subsequently, after removing any unbound substances, a biotin-conjugated antibody specific for B7-H4 was added to the wells for one hour at 37°C. After washing three times, 100 μL of avidin conjugated Horseradish Peroxidase (HRP) were added for 1 h at 37°C. Subsequently, after washing five times, 90 μL of substrate solution containing TMB were added for 15-30 minutes at 37°C, protected from light. Color development was stopped by the addition of 50 µl stop solution to each well and the degree of enzymatic turnover of the substrate was investigated by dual-wavelength absorbance measurement at 450 and 620 nm as a reference wavelength within 5 minutes using an ELISA reader (TECAN, Model Sunrise; Austria GmbH, Grodig, Austria) and the data analysis software Magellan™ (TECAN, Mannedorf, Switzerland). To quantify the blood serum concentration levels of B7-H4, a non-linear regression model (4-parameter Marquardt) was used with a log/lin type of graph according to the manufacturer’s instructions. The observed absorbance was directly proportional to the concentration level of sB7-H4 in the samples, which was calculated from the calibration curve. The sB7-H4 serum levels are expressed in ng/mL according to the established standard curve (detection range: 7.8 - 500 ng/mL). The minimum detectable dose of sB7-H4 was typically less than 1.95 ng/mL. The lower limit of detection (LLD) was defined as the lowest protein concentration that could be differentiated from zero. Intra-assay variation was <8%, while inter-assay variation was <10%.

### Selection and Detection of CTCs

The selection, detection, characterization and data evaluation of epithelial CTCs and CTCs in EMT in EOC has been published in detail by our group (5, 16–18).

10 ml blood was collected in EDTA tubes (Sarstedt & Co.) from each patient and processed within 4h for the enrichment of CTCs and subsequent expression analysis according to the Adnatest OvarianCancer (QIAGEN, Hilden, Germany). Briefly, CTCs were immunomagnetically selected using the AdnaTests OvarianCancerSelect and EMT-1Select. Subsequently, RNA was isolated and gene expression analysis was performed by reverse-transcription (RT) and multiplex RT-PCR detecting a) EpCAM, MUC-1, and CA-125 (AdnaTest OvarianCancerDetect; ERCC1-transcripts were investigated in a separate approach by singleplex RT-PCR) and b) PIK3CA, AKT2 and TWIST (AdnaTest EMT).

All AdnaTests were purchased from QIAGEN (Hilden, Germany). Positivity for each CTC-subtype was defined by the detection of at least one of the transcripts of each marker panel, respectively. A patient was defined as CTC-positive if epithelial CTCs and/or CTCs in EMT were detected.

### Statistical Analysis

The distribution of sB7-H4 in each study group was different from normal. Descriptive statistics were computed and reported as median with interquartile range (IQR) or frequency counts (%). The differences between two groups were defined by Mann-Whitney U test. Differences in frequency counts were analyzed using the Chi-squared test. Kaplan-Meier analysis was performed to analyze DFS and overall survival (OS) probabilities. The difference between survival curves was assessed by using the log rank test. Hazard ratios (HR) with corresponding 95%-confidence intervals (95%-CI) were calculated by using Cox proportional hazards regression. The Spearman rank correlation coefficient was calculated to examine whether the sB7-H4 levels were monotonically related to CA12-5 serum levels. All analyses were performed using the MedCalc version 17.9.7 (MedCalc Software bvba, Ostend, Belgium).

## Results

The median age of the patients at primary diagnosis was 60 years (37 - 82, SD 11.7). The clinical data of patients included in the study are shown in **Table 1**. The majority of patients were in Figo stage III (71.3%) compared with stage IV (28.2%). High-grade tumors (G3) were found in 58 (70.7%) patients whereas Grade 1 and 2 in 24 (29.3%) of patients. 40 (62.5%) of patients had positive lymph-nodes and 24 (37.5%) negative nodal-status. When stratifying according to resection status 39 (48.1%) had complete resection (R0) compared with 42 (51.9%) patients with incomplete resection. In 48 (62.3%) patients a recurrent disease was diagnosed (**Table 1**). Among patients with platinum-resistant disease 15 (75%) had positive nodal-status, compared with 5 (25%) with negative nodal-status. In FIGO IV stage 12 (75%) patients and in FIGO III stage 28 (70%) had lymph-nodes metastasis. Follow-up was calculated from the time of diagnosis to the date of last follow-up information or death. Median follow-up time was 35.5 months (0 - 139, SD 28.1). Analyzing only patients who had died, medium time to death was 25 months (0 - 75, SD 22.8). Medium DFS was 27 months (0 - 130, SD 23.9). Patients with an OS ≤ 4 months were excluded from survival analyses in order to rule out early, surgery related deaths (n=12).

### Association of sB7-H4 Concentration Levels, CTCs, and Clinicopathological Characteristics

sB7-H4 was detectable in 12/85 (14.1%) patients, CTCs in 28/85 (32.9%) patients and simultaneous presence of CTCs and sB7-H4 was found in 6/85 (7%) patients.

Epithelial CTCs were found in 10/85 (11.7%) patients and EMT-like CTCs in 18/85 (21.2%) patients. In 5/85 (5.9%) patients, expression of both epithelial and EMT-like CTCs subtypes was found. sB7-H4 positivity was significantly more common in patients without confirmed (pN0) spread to lymph nodes (25%) compared to patients with histologically confirmed (pN1) metastases in lymph nodes (12.5%, p=0.04). Moreover, sB7-H4 presence in serum was significantly associated with platinum resistance (p=0.03). CTCs presence in blood was slightly higher in platin resistant patients but the difference was not significant. There were no differences in sB7-H4 levels in serum according to clinicopathological parameters. Associations between sB7-H4 concentration levels, CTCs and clinicopathological characteristics including FIGO stage, grading, nodal status, sensibility to platin agents and resection status are presented in **Table 2**.

**Table 2:** Associations of sB7-H4 blood serum levels, sB7-H4 presence in serum and CTCs to clinicopathological parameters.

Total n = 85	sB7-H4 ng/mL levels in serum (IQR)	p-value	sB7-H4 presence in serum (%)	p-value	CTCs presence in blood (%)	p-value
FIGO stage						
III	32.89 (5.41 - 71.9)	0.79	8 (13.1)	0.19	17 (27.8)	**0.11**
IV	31.05 (22.5 - 44.47)		6 (25)		11 (45.8)	
Grading						
I and II	30.37 (22.5 - 92.97)	0.58	4 (16.6)	0.4	11 (45.83)	0.7
III	31.73 (5.01 - 49.29)		11 (18.9)		17 (29.3)	
Lymph node status						
N0	21.79 (6.2 - 31.73)	0.24	6 (25)	0.04	3 (12.5)	0.18
N1	44.58 (17.49 - 87.6)		5 (12.5)		16 (40)	
Platinum sensitivity						
Sensitive	33.48 (4.98 - 47.76)	0.25	8 (16)	0.03	15 (30)	0.08
Resistant	31.05 (21.08 - 82.23)		6 (66.6)		13 (56.5) 23	
Resection status						
Complete resection	25.72 (5.41 - 41.28)	0.43	8 (20.5)	0.4	10 (25.6)	0.15
Incomplete resection	44.58 (22.5 - 82.23)		6 (14.3)		17 (40.47)	

Comparisons between sB7-H4 serum levels were analyzed using Mann-Whitney U test, sB7-H4- and CTC-presence using chi-square test.

### Impact of sB7-H4 and CTC Presence on Patients’ Survival

**Figure 1:**
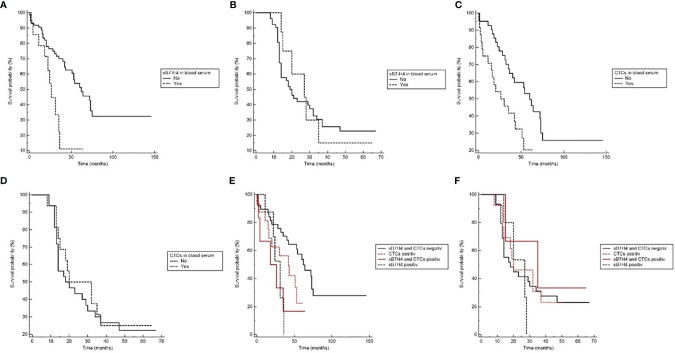
The Kaplan-Meier plots showing prognostic relevance of sB7-H4 and CTC detection in blood serum. **(A)** Overall survival (OS) with regard to sB7-H4 detection (p=0.004). **(B)** Disease-free survival (DFS) with regard to sB7-H4 detection (ns). **(C)** Overall survival (OS) with regard to CTC detection (Log-rank p=0.008). **(D)** Disease-free survival (DFS) with regard to CTC detection (ns). **(E)** Overall survival (OS) for the different combinations of sB7-H4 and CTCs (Log-rank p=0.03). **(F)** Disease-free survival (DFS) for the different combinations of sB7-H4 and CTCs (ns).

Comparison between patients with detectable sB7-H4 serum-levels and no detectable serum levels revealed a statistically significant effect on OS. Patients with detectable sB7-H4 in blood serum showed a significantly shorter survival time than patients with no sB7-H4 detection (HR 4.42, 95% CI 1.61 – 12.13; p = 0.004). No effect on DFS could be observed (**Figure 1**).

Patients with detectable CTCs showed a significantly shorter survival time than patients in whom no CTCs were detected (HR 2.63, 95% CI 1.28 - 5.39; p = 0.008). No effect on DFS could be observed (**Figure 1**).

Univariate Cox regression analysis indicated that the presence of sB7-H4 and CTCs influenced the prognosis of our patients. Patients presenting with enhanced sB7-H4 levels and CTCs had a significantly worse OS in comparison with those patients with no detectable sB7-H4 and CTCs (HR 2.8, 95% CI 11.34 – 5.82; p=0.006 and HR 2.32, 95% CI 1.22 – 4.41; p=0.01, respectively). The clinical factors associated with prognosis including platinum-sensitivity, resection status, FIGO stadium and lymph node status also correlated with OS, however, apart from platinum-sensitivity, no effect on DFS could be observed (**Table 3**).

**Table 3:** Results of univariate Cox regression analysis of patients with advanced EOC.

	OS		DFS	
	HR (95% CI)	p-value	HR (95% CI)	p-value
sB7-H4 in serum				
no	1	0.006	1	0.82
yes	2.8 (1.34 – 5.82)		0.9 (0.38 – 2.14)	
CTCs in blood				
no	1	0.01	1	0.4
yes	2.32 (1.22 – 4.41)		0.85 (0.42 – 1.69)	
Residual tumor				
no	1	0.01	1	0.16
yes	2.13 (1.17 – 3.88)		1.54 (0.84 – 2.82)	
FIGO stage				
III	1	0.12	1	0.65
IV	1.63 (0.88 – 3.04)		0.85 (0.42 – 1.72)	
Grade				
I/II	1	0.78	1	0.79
III	1.09 (0.59 – 2.03)		1.09 (0.56 – 2.08)	
Lymph node status				
N0	1	0.1	1	0.47
N1	1.95 (0.87 – 4.39)		1.35 (0.6 – 3.03)	
Platinum sensitivity				
Sensitive	1	<0.0001	1	0.03
Resistant	7.03 (4.1 – 12.08)		10.8 (1.26 – 92.74)	

CI, confidence interval; HR, hazard ratio.

Multivariate Cox regression analysis including resection status, FIGO stage, grade, lymph node status, platinum-sensitivity, CTC detection and sB7-H4 presence indicated that sB7-H4 and CTCs presence were independent predictive factor for OS (HR 3.57, 95% CI 1.23 - 10.36; p=0.02 and HR 4.31, 95% CI 1.53 - 12.17; p=0.006, respectively). sB7-H4 or CTC presence did not significantly influence the adjusted hazard on developing recurrent disease (**Table 4**).

**Table 4:** Effect of sB7-H4 and CTCs presence on OS and DFS after correcting for unfavorable prognostic factors in cox proportional hazards regression model.

	OS		DFS	
	HR (95% CI)	p-value	HR (95% CI)	p-value
sB7-H4 in serum				
no	1	0.02	1	0.74
yes	3.57 (1.23 -10.36)		1.3 (0.27 – 6.08)	
CTCs in blood				
no	1	0.006	1	0.35
yes	4.31 (1.53 - 12.17)		0.59 (0.19 – 1.78)	
Residual tumor				
no	1	0.3	1	0.65
yes	1.53 (0.61 – 3.83)		1.27 (0.45 – 3.59)	
FIGO stage				
III	1	0.16	1	0.86
IV	0.47 (0.16 - 1.36)		0.89 (0.23 – 3.4)	
Grade				
I/II	1	0.22	1	0.4
III	0.58 (0.25 - 1.38)		1.5 (0.57 – 3.93)	
Lymph node status				
N0	1	0.58	1	0.73
N1	1.31 (0.5 – 3.4)		1.21 (0.41 – 3.54)	
Platinum sensitivity				
Sensitive	1	<0.0001	1	<0.0001
Resistant	7.24 (3.03 – 17.33)		59.1 (4.52 – 771.97)	

CI, confidence interval; HR, hazard ratio.

### sB7-H4 and CTCs

22 out of 59 patients (37.2%) with normal sB7-H4 levels had detectable CTCs, whereas in the group of patients with detectable sB7-H4 levels, 6 out of 12 (50%) patients were positive for CTCs. However, this effect failed to reach statistical significance (p = 0.41). An overview on the presence of sB/-H4 and CTCs is given in (**Table 5**).

**Table 5:** Presence of sB7-H4 and CTCs in blood serum in patients with advanced EOC.

	sB7-H4 detectable	sB7-H4 not detectable
CTCs detectable in blood serum	6	22
CTCs not detectable in blood serum	6	37

There were no differences in sB7-H4 serum levels between patients harboring CTCs (median 31.05, IQR13.64-63.46) or not (median 27.29, IQR 3.76-50.83) (p=0.69).

Patients with detectable sB7-H4 levels and CTCs showed a significantly shorter survival time than patients with normal sB7-H4 levels and no detectable CTCs (p = 0.03). No effects on DFS could be observed (**Figure 1**).

## Discussion

Our study found that detection of sB7-H4 and CTCs in blood was associated with a worse prognosis of patients with advanced EOC. However, there was no association between sB7-H4 and CTC presence. Current literature supports that high expression of B7-H4 was negatively correlated with survival outcome in OC, suggesting that B7-H4 plays an essential role in poor prognosis (16). However in a meta-analysis from Ye et al., membrane-bound B7-H4 was not related to patients’ clinicopathologic characteristics (19). These results are consistent with our findings. However, it is not clear whether serum sB7-H4 levels reflect the expression of B7-H4 in tumor tissue. Similarly to the previous study for sB7-H4 and EOC (13), we found that serum sB7-H4 predicted a worse prognosis (being a predictive factor for OS), independent of clinical parameters known for worse outcomes.

In our study, patients with detectable sB7-H4 and CTCs in blood showed significantly worse survival. The function and source of sB7-H4 are still unknown but some authors have demonstrated that sB7-H4 secretion is promoted under inflammatory conditions (20–22) and in EOC, B7-H4 has been associated with a pro-inflammatory tumor microenvironment (23). On the other hand, the neutrophile-derived inflammatory response was shown to interact with CTCs expanding their metastatic potential (24). These data could be a possible explanation of the worse prognosis of EOC patients showing the simultaneous presence of sB7-H4 and CTCs in the bloodstream.

In our study sB7-H4 was found more in patients without confirmed spread to lymph-nodes, which could suggest its association with better outcome. This fact may be explained due to early way of dissemination in EOC, which is primary into the peritoneal cavity and not into lymph-nodes. On the contrary, sB7-H4 presence was associated with platinum resistance, which is one of the most important factors connected with worse prognosis. These findings suggest that the tumor biology, rather than tumor load are responsible for sB7-H4 presence in advanced EOC.

The interaction between tumor cells and their microenvironment plays a crucial role in cancer development (25). Current data on the relationship between T-Cell regulators and CTCs in EOC are limited. However, strong evidence suggests a direct interaction between CTCs and the immune system in the peripheral blood. Mego et al. showed that patients with inflammatory BC harboring CTCs had an impaired adaptive immunity (26). In the current study, both, the presence of CTCs and sB7-H4 were associated with a worse prognosis. Postulating a function of B7-H4 as part of the tumor’s immune-escape mechanisms, it seems plausible that an increased expression of the immune-checkpoint molecule increases the risk of tumor spread to the blood, indicating poor outcome. This reflects higher sB7-H4 levels in patients with positive CTCs. This is in line with our recent findings analyzing soluble PD-L1 and PD-L2 in EOC patients (9). However, we did not find any association between sB7-H4 and CTCs presence in serum. Keeping in mind that sB7-H4 and CTCs were detectable in only 15 and 28 patients, respectively, this lacking association might well be attributable to the small sample size. Nevertheless, the potential association between CTCs and soluble and membrane T-cells regulators should be further investigated since checkpoint-inhibitors showed promising results in therapy management of advanced EOC (27, 28).

Expression of immune-checkpoints on CTCs can be used as a prognostic indicator and to monitor and evaluate the efficacy of immune checkpoint inhibitors (29–33). For instance, in patients diagnosed with breast cancer, PD-L1-positive CTCs have a prognostic predictive value (33). Our data suggest that the simultaneous detection of sB7-H4 and CTCs in blood serum might have prognostic value.

The role of immune-checkpoint inhibition in the therapy of advanced EOC has been intensively investigated in recent years. It was reported that B7-H4/CD3-bispecific antibodies might be a therapeutic agent against B7-H4-expressing tumors (34). One of the biggest issues concerning checkpoint-inhibitor therapy is the identification of factors predicting therapy response. Therefore, it is crucial to identify markers that can predict the possible response to this treatment option. The presence of sB7-H4 could be a predictive marker for immunotherapy, targeting T-cells as shown by Azuma (35, 36).

There are some limitations to this study. Unfortunately, we do not have results for B7-H4 on tumor tissue for comparison studies, which should be addressed in future work. In addition, we presented a retrospective study with limited sample size. Nevertheless, our data suggest that sB7-H4 and CTCs should be considered potentially useful markers, predicting the prognosis in advanced EOC. A deeper understanding of this interaction may provide an opportunity for new therapeutic strategies.

## Data Availability Statement

The raw data supporting the conclusions of this article will be made available by the authors, without undue reservation.

## Ethics Statement

The studies involving human participants were reviewed and approved by Ethics committee of the University of Duisburg-Essen (Essen 05-2870 and 17-7859). The patients/participants provided their written informed consent to participate in this study.

## Author Contributions

PM: study design, data acquisition, providing of blood and tissue samples, statistical analysis, and manuscript writing. RK: providing of blood and tissue samples, and manuscript editing. SK-B: study design, data acquisition, laboratory analyses, providing of blood and tissue samples, and manuscript editing. PB: study design, data acquisition, providing of blood and tissue samples, statistical analysis, and manuscript writing. All authors contributed to the article and approved the submitted version.

## Conflict of Interest

SK-B is a consultant for Qiagen.

The remaining authors declare that the research was conducted in the absence of any commercial or financial relationships that could be construed as a potential conflict of interest.

## Publisher’s Note

All claims expressed in this article are solely those of the authors and do not necessarily represent those of their affiliated organizations, or those of the publisher, the editors and the reviewers. Any product that may be evaluated in this article, or claim that may be made by its manufacturer, is not guaranteed or endorsed by the publisher.
